# Immunity-Based Diagnosis for a Motherboard

**DOI:** 10.3390/s110404462

**Published:** 2011-04-18

**Authors:** Haruki Shida, Takeshi Okamoto, Yoshiteru Ishida

**Affiliations:** 1 Department of Information Network and Communication, Kanagawa Institute of Technology 1030, Shimo-ogino, Atsugi, Kanagawa 243-0292, Japan; E-Mail: haruki.shida@gmail.com; 2 Department of Knowledge-Based Information Engineering, Toyohashi University of Technology, 1-1, Tempaku, Toyohashi, Aichi 441-8580, Japan; E-Mail: ishida@cs.tut.ac.jp

**Keywords:** immunity-based system, fault diagnosis, sensor, motherboard, immune network

## Abstract

We have utilized immunity-based diagnosis to detect abnormal behavior of components on a motherboard. The immunity-based diagnostic model monitors voltages of some components, CPU temperatures, and fan speeds. We simulated abnormal behaviors of some components on the motherboard, and we utilized the immunity-based diagnostic model to evaluate motherboard sensors in two experiments. These experiments showed that the immunity-based diagnostic model was an effective method for detecting abnormal behavior of components on the motherboard.

## 1. Introduction

The technology of cloud computing has become prevalent, and the demand for data centers that provide such cloud computing has increased. Each server in the data center must be highly available for data processing and data transmission. To maintain system availability, it is important to detect equipment abnormalities during their early stages, before system failure. The simplest way of diagnosing abnormalities consists of evaluating each component individually by comparing the output value of its sensor with a predetermined threshold value. However, it is difficult to identify the abnormal component using this method [1].

Another method of diagnosis uses an immunity-based diagnostic model [2–7], which is derived primarily from the concept of an immune system [8]. In the biological immune systems, each immune cell can test other immune cells and can be tested by other immune cells, and protects against disease by identifying and eliminating nonself entities (*i.e.*, pathogens). Similarly, in our diagnostic model, mutual tests are performed among nodes (*i.e.*, sensors), and this protects against system failure by identifying abnormal nodes. The features of our diagnostic model are similar to the features of the biological immune systems, therefore, the diagnostic model is called the immunity-based diagnostic model. This diagnostic model has been applied to node fault diagnosis in processing plants [9], to self-monitoring/self-repairing in distributed intrusion detection systems [3], and to sensor-based diagnostics for automobile engines [4]. This paper reports on the use of an immunity-based diagnostic model for detecting the abnormal behavior of components on a motherboard, including CPUs, memories, chipsets and Fans.

## 2. Embedded Sensors on the Motherboard

Since a motherboard has multiple sensors, including voltage, temperature, and fan speed sensors, abnormalities on the motherboard can be detected by monitoring these sensors. We therefore used sensor output values for diagnosis of the motherboard.

We collected sensor output values on a server from July 27th to September 18th. The specifications of the server are shown in Table 1. The average air temperature during that period was 25.3 °C, ranging from 20.1 °C to 32.8 °C. Data were collected using lm_sensors, a hardware health monitoring package for Linux that allows information to be obtained from temperature, voltage, and fan speed sensors.

We collected the output values from all 29 sensors on the motherboard, from which we calculated the correlation coefficients of all sensors. The correlation coefficient *C* of a set of sensor data (*x*, *y*) = {(*x_i_*, *y_i_*) (*i* = 1,2, …*n*)} is given by the following equation:

(1) $$C=\frac{\sum_{i=1}^{n}(x_{i}-\bar{x})(y_{i}-\bar{y})}{\sqrt{\sum_{i=1}^{n}{\left(x_{i}-\bar{x}\right)}^{2}}\sqrt{\sum_{i=1}^{n}{\left(y-\bar{y}\right)}^{2}}}$$

where:

(2) $$\bar{x}=\frac{1}{n}\sum_{i=1}^{n}x_{i},\bar{y}=\frac{1}{n}\sum_{i=1}^{n}y_{i}$$

We observed correlations between five sensors (Table 2), and these five sensors are easy to assume that the test cases for evaluation. Therefore, we used these five sensors for evaluation.

## 3. Immunity-Based Diagnostic Model

The immunity-based diagnostic model has the features of a dynamic network [7], in which diagnoses are performed by mutually testing nodes, *i.e.*, sensors, and by dynamically propagating their active states. In this paper, the targets of the immunity-based diagnosis are components with a sensor embedded on a motherboard. Each sensor can test linked sensors and can be tested by linked sensors. Each sensor is assigned a state variable *R_i_* indicating its credibility.

The initial value of credibility *R_i_* (0) is 1. The aim of the diagnosis is to decrease the credibility of all the abnormal sensors. If the credibility of a sensor is less than a threshold value, the sensor is considered abnormal in this model.

When the value of credibility *R_i_* is between 0 and 1, the model is called a *gray model,* reflecting the ambiguous nature of credibility. The *gray model* is formulized by the equation:

(3) $$\frac{dr_{i}(t)}{\mathrm{dt}}=\sum_{j}T_{\mathrm{ji}}^{+}R_{j}(t)-r_{i}(t)$$

where:

(4) $$R_{i}=\frac{1}{1+\text{exp}\;(-r_{i}(t))}$$

(5) $$T_{\mathrm{ij}}^{+}=\{\begin{array}{c} T_{\mathrm{ij}}+T_{\mathrm{ji}}-1,\;\text{if one of evaluation from i to j or j to i exists},\\ 0,\;\text{if neither evaluation from i to j nor j}\;\text{to i exists}, \end{array}$$

(6) $$T_{\mathrm{ij}}=\{\begin{array}{c} 1,\;\text{if a balance formula between sensors }i\;\text{and }j\;\text{is satisfied},\\ -1,\;\text{if a balance formula between sensors}\;i\;\text{and }j\;\text{is not satisfied},\\ 0,\;\text{if there is}\;\text{no balance formula between sensors }i\;\text{and}\;j. \end{array}$$

Equation (3) controls the commitment of the node by determining the variable *r_i_*(*t*) based on the evaluations to and from the node *i* and the active/inactive state of the evaluating and being evaluated nodes *j*. In the right-hand side of Equation (3), the first term is the sum of evaluations from other nodes for node *i*. The second term is an inhibition term that maintains ambiguous states of credibility. Activeness of each node *i* will be expressed by a continuous time dependent variable *r_i_* ∈ {–∞, ∞} or its normalization *R_i_* ∈ [0,1]; *Ri* = 1 for fully active (*R_i_* = 0 for fully inactive).

**Figure 1.**
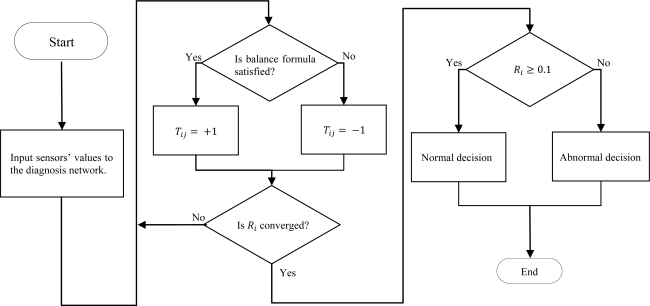
Flowchart of the diagnostic model.

In this model, equilibrium points satisfy the equation $r_{i}(t)=\sum {}_{j}T_{ji}^{+}R_{j}(t)$. Thus *R_i_* monotonically reflects the value of $\sum {}_{j}T_{\mathrm{ji}}^{+}R_{j}(t)$. If $\sum {}_{j}T_{\mathrm{ji}}^{+}R_{j}(t)$ is close to 0, then *R_i_* is close to 0.5. The balance formulas are shown in Table 3. We determined the balance formulas by calculating the relationships of the output value of the sensors by trial and error. The flowchart of the diagnostic model is shown in Figure 1.

## 4. Evaluations of Immunity-Based Diagnosis of the Motherboard

We evaluated the immunity-based diagnostic model for motherboard sensors in two experiments. In the first experiment, we compared two diagnostic models: a standalone diagnostic model and a mutual diagnostic model, *i.e.*, an immunity-based diagnostic model. In the second experiment, we compared two networks in the immunity-based diagnostic model: a fully-connected network and a correlation-based network. We determined the normal ranges by calculating the balance formulas. Table 4 shows the normal ranges. Each evaluation was based on the four test cases shown in Table 5, and the value of test cases was based on the range of sensor output values shown in Table 2 and the normal ranges shown in Table 4.

The test cases in 1 and 2 assumed that the speed of Fan5 was largely out of the range shown in Table 2. A significant decrease in fan speed would therefore cause the CPU temperature to rise, with the overheated CPU causing the server to crash. Conversely, a significant increase in fan speed would waste power and decrease the life span of the fan. In addition, the output values of the sensors were largely out of the range shown in Table 4. Therefore, we determined that the test cases of 1 and 2 are abnormal.

The test cases of 3 and 4 assumed that the output values of the sensors were slightly out of the range shown in Table 2. The test case of 3 assumed that the speed of Fan5 was slightly higher than that of Table 2, but that Fan5 was not abnormal. The test case of 4 assumed that the temperature of CPU1 was slightly higher than that of Table 2, but that CPU1 was not abnormal. Temperatures outside the range are not always abnormal, because these temperatures depend on room temperature. For example, maximum of temperature differences is 12.7 °C. In addition, the output values of the sensors were inside of the range shown in Table 4. Therefore, we determined that the test cases of 3 and 4 are normal.

### 4.1. Stand Alone *vs.* Mutual Diagnosis

We evaluated a standalone diagnosis and a mutual diagnosis. According to the standalone diagnosis, a component is considered abnormal if the sensor output value is outside the range shown in Table 2. In contrast, mutual diagnosis uses the immunity-based diagnostic model.

Tables 6 and 7 show the results of the standalone and mutual diagnoses, respectively. In Table 6, a credibility of 0 indicates that the output value was not within range, *i.e.*, it was abnormal, whereas a credibility of 1 indicates that the output value was within range, *i.e.*, it was normal. In Table 7 the credibility corresponds to *R_i_* of Equation (2), *i.e.*, it expresses the probability that component *i* is normal. We assumed that a component on the motherboard was abnormal if its credibility was less than 0.1. This threshold value is an empirical value by trial and error. A diagnosis of “X” indicates an abnormality, whereas a diagnosis of “O” indicates an absence of abnormality. An accuracy of “O” indicates a correct decision, an accuracy of “X” indicates an incorrect decision, and an accuracy of “P” indicates that the diagnostic model could not identify the abnormal component, although it detected multiple abnormalities.

The standalone diagnostic model detected abnormalities in all test cases, because all test cases have values out of the range. In test cases 1 and 2, the standalone diagnostic model failed to identify the abnormal component. This model also misdiagnosed test cases 3 and 4, judging them abnormal since the output values were slightly out of the range. In contrast, the mutual diagnosis model identified the abnormal Fan in test case 2 since only the credibility of Fan5 was 0.00. In test case 3, the mutual diagnosis made a correct decision. Consequently, the mutual diagnosis model is more accurate than the standalone diagnosis model.

### 4.2. Fully-Connected Network *vs.* Correlation-Based Network

The immunity-based diagnostic model contains a network for mutually testing the credibility of nodes. In the above section, the network of the immunity-based diagnostic model was fully-connected, with each sensor connected to all other sensors, and each sensor mutually tested by all other sensors. A fully-connected network can include some connections between sensors with weakly correlated output values. These connections may be unreliable for mutually testing the credibility of their sensors. Therefore, we removed such connections from a fully-connected network, forming a correlation-based network.

**Figure 2.**
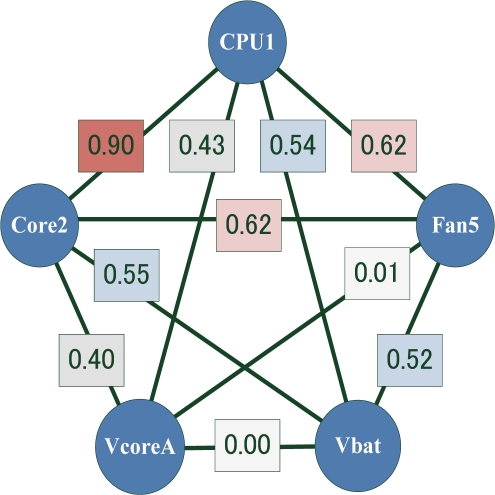
Correlation coefficients among five sensors.

**Figure 3.**
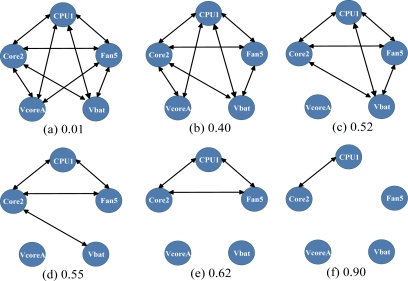
Correlation-based networks for thresholds of (**a**) 0.01, (**b**) 0.40, (**c**) 0.52, (**d**) 0.55, (**e**) 0.62, and (**f**) 0.90.

We used the immunity-based diagnostic model to evaluate two network models, a fully-connected network and a correlation-based network. Figure 2 shows the correlation coefficients among the 5 sensors in Table 2. Any pair of sensors with a correlation greater than a threshold value was defined as connected. In this experiment, we built correlation-based networks for all the thresholds, using the correlation coefficients shown in Figure 2. Typical correlation-based networks are shown in Figure 3.

All test cases were the same as those in Table 5. Table 8 shows the results of correlation-based networks. A network with a threshold less than 0.01 was identical to a fully-connected network, whereas a network with a threshold greater than 0.90 had no connection between any pair of sensors, *i.e.*, a diagnostic model with a threshold greater than 0.90 was identical to a stand alone diagnostic model. These diagnostic models were evaluated in the previous section.

In Table 8(A) the diagnostic models with thresholds of 0.01 misidentified the normal CPU1 in test cases 1 and 4. In Table 8(B), the diagnostic models with thresholds of 0.40 misidentified the normal CPU1 in test cases 1, 2 and 4. In Table 8(C), the diagnostic model with a threshold of 0.52 identified the abnormal Fan in test cases 1 and 2, and did not falsely identify an abnormality in test case 3, but misidentified the abnormal CPU1 in test case 4 as normal. In Table 8(D,E), the diagnostic models with thresholds of 0.55 and 0.62 correctly identified the abnormal Fan in test cases 1 and 2 and did not falsely identify abnormalities in test cases 3 and 4. In Table 8(F), the diagnostic model with a threshold of 0.90 identified only test case 3, because the abnormal sensor of Fan5 was isolated from the correlation-based network. This diagnostic model could not diagnose the isolated sensors, because the credibility of each was always 0.50.

**Figure 4.**
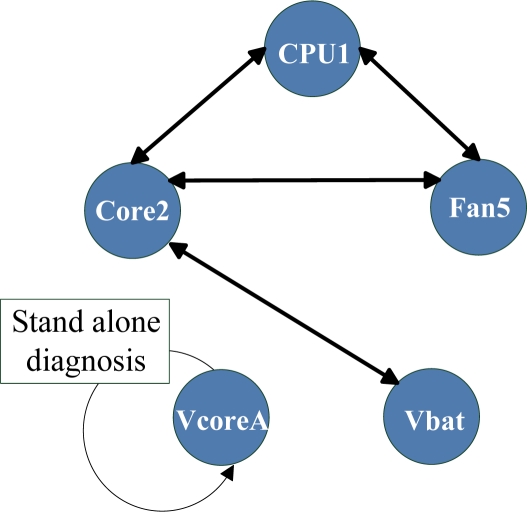
Example of a hybrid diagnostic model with a threshold of 0.55.

Even networks with the best thresholds, of 0.55 and 0.62, have isolated sensors of VcoreA and Vbat. The sensor output values of VcoreA and Vbat were approximately constant over time, *i.e.*, their standard deviations were very small (Table 2), such that the standalone diagnostic model would correctly detect their abnormalities. Therefore, we applied standalone diagnosis only to these isolated sensors (Figure 4). In other words, we use a hybrid diagnosis model, using both standalone and immunity-based diagnosis. Sensors on the correlation network were diagnosed by the immunity-based diagnostic model, and isolated sensors were diagnosed by the stand alone diagnostic model.

### 4.3. Discussions of Multiple Diagnostic Networks

**Figure 5.**
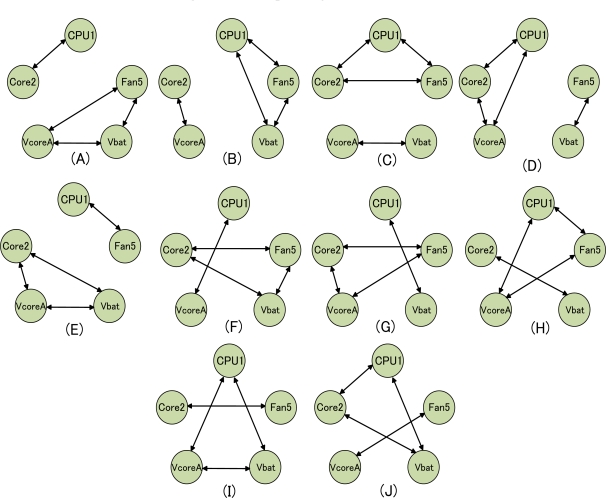
Multiple diagnostic networks.

We hypothesized that utilizing multiple diagnostic networks, in which isolated nodes are connected to a network or another isolated node, would improve diagnostic accuracy. All combinations of the multiple networks used for immunity-based diagnosis are shown in Figure 5. Each evaluation was based on the four test cases shown in Table 5. The diagnostic accuracy of all multiple networks is shown in Table 9. In Table 9, a diagnostic accuracy of “P” indicates that the diagnostic model could not identify the abnormal component, although it detected multiple abnormalities.

We found that diagnostic models (A), (C), (F) and (G) made correct decisions, whereas the other diagnostic models made incorrect decisions. In test cases 1, 2 and 3, each of the diagnostic networks (A), (C), (F) and (G) consisted of 3 sensors including Fan5. In contrast, the other diagnostic networks either consisted of 2 sensors including Fan5 or were weakly correlated networks. In test case 4, all diagnostic networks other than (B) and (I) showed results similar to those of CPU1.

For example, Table 10 shows the successful results of diagnostic network (C), and Table 11 shows the unsuccessful results of diagnostic network (I).

The diagnostic model in Table 11 misidentified the abnormal Fan5 in test case 2 and test case 3. These results indicate that the diagnostic network consisting of 3 sensors is more accurate than the diagnostic network consisting of two sensors. In test case 4 of Table 11, the diagnostic network misidentified the normal CPU1 due to a weak correlation network shown in Figure 2, although CPU1 belongs to the diagnostic network consisting of three sensors. These results indicate that the strong correlated diagnostic network is more accurate than the strong weakly correlated diagnostic network. Therefore, these experiments showed that diagnostic accuracy depends on the number of sensors in the diagnostic network (*i.e.*, the size of diagnostic network) and the correlation between sensors of network.

## 5. Conclusions

We have applied immunity-based diagnosis to the detection of abnormal behaviors of components on a motherboard. We simulated the abnormal behaviors of some components on the motherboard, and we evaluated the ability of this model to diagnose abnormalities of components of motherboard sensors by two experiments. In the first experiment, which compared an immunity-based with a stand-alone diagnostic model, we found that the immunity-based diagnostic model outperformed the standalone diagnostic model. In the second experiment, which compared a fully-connected network with a correlation-based network for mutually testing the credibility of sensors, and we found that the correlation-based network improved the diagnosis accuracy in all test cases. In addition, we evaluated all the combinations of the diagnostic networks, and we showed that diagnostic accuracy depends on the size of the network and the correlation between nodes of the network. At the same time, we showed that the immunity-based diagnostic model with multiple diagnostic networks was an effective method for detecting abnormal behavior of components on the motherboard.

In addition, we utilized a hybrid model, consisting of the standalone and immunity-based diagnostic models, to diagnose nodes connected to the network, as well as nodes isolated from the network. The accuracy of hybrid diagnosis, however, depends on the stand alone diagnosis for the isolated nodes. In future, we will attempt to improve the accuracy of diagnosis of isolated nodes.

## Figures and Tables

**Table 1.** Server specification.

Motherboard	Supermicro^®^ X7DVL-I
OS	Debian GUN/Linux 5.0
Kernel	2.6.26-2-amd64
Module	lm-sensors version 3.0.2 with libesensors version 3.0.2
CPU	Intel^®^ Xeon E5410 2.33GHz×2
Power supply	Thermaltake Toughpower 700w
Fan	XFan model: RDM8025B×2, Gantle Typhoon D0925C12B2AP×2, ADDA CFX-120S

**Table 2.** Sensors used for evaluation and the range of sensor output values.

**Sensor**	**Component**	**Range**	**Mean**	**Standard deviation**

CPU1	CPU temperature	11.00–48.00(°C)	18.68	4.550
Core2	Core2 temperature	35.00–72.00(°C)	42.79	4.450
VcoreA	CoreA voltage	1.11–1.19(V)	1.121	0.007
Vbat	Internal battery voltage	3.23–3.26(V)	3.237	0.009
Fan5	Fan speed	1,012–1,044(RPM)	1034	5.021

**Table 3.** Balance formulas between sensors.

**Sensor**	**Balance formula**

CPU1-Core2	|CPU1-Core2| ≤ 26
CPU1-VCoreA	|CPU1-VCoreA × 25| ≤ 20
CPU1-Vbat	|CPU1-Vbat × 9| ≤ 18
CPU1-Fan5	|CPU1-Fan5/34| ≤ 18
Core2-VCoreA	|Core2-VcoreA × 45.5| ≤ 28
Core2-Vbat	|Core2-Vbat × 16| ≤ 20
Core2-Fan5	|Core2-Fan5/19| ≤ 21
VCoreA-Vbat	|VCoreA-Vbat/2.8| ≤ 0.05
VCoreA-Fan5	|VCoreA-Fan5/893| ≤ 0.07
Vbat-Fan5	|Vbat-Fan5/316| ≤ 0.07

**Table 4.** Normal ranges derived from the balance formulas.

**Sensor**	**Normal range**

CPU1	4.75–78.16(°C)
Core2	31.68–73.34(°C)
VcoreA	0.99–1.31(V)
Vbat	2.52–3.96(V)
Fan5	821.56–1,232.34(RPM)

**Table 5.** Test cases.

**Case**		**Sensor output value**					**State**
		**CPU1**	**Core2**	**VcoreA**	**Vbat**	**Fan5**	

1	Fan speed is very low.	70	65	1.12	3.23	200	Abnormal
2	Fan speed is very high.	9	35	1.12	3.23	2,000	Abnormal
3	Fan speed is slightly high.	14	35	1.12	3.23	1,050	Normal
4	CPU temperature is slightly high.	50	60	1.12	3.23	1,020	Normal

**Table 6.** Results of the stand alone diagnosis.

**Test case**	**Credibility**					**Decision**	**Accuracy**
	**CPU1**	**Core2**	**VcoreA**	**Vbat**	**Fan5**		

1	0	1	1	1	0	X	P
2	0	1	1	1	0	X	P
3	1	1	1	1	0	X	X
4	0	1	1	1	1	X	X

**Table 7.** Results of the mutual diagnosis.

**Test case**	**Credibility**					**Decision**	**Accuracy**
	**CPU1**	**Core2**	**VcoreA**	**Vbat**	**Fan5**		

1	0.00	0.83	0.78	0.78	0.00	X	P
2	0.73	0.99	0.99	0.73	0.00	X	O
3	0.99	0.91	0.99	0.99	0.99	O	O
4	0.00	1.00	1.00	1.00	1.00	X	X

**Table 8.** (**A**) Results of a correlation-based network with a threshold of 0.01.

**Test case**	**Credibility**					**Decision**	**Accuracy**
	**CPU1**	**Core2**	**VcoreA**	**Vbat**	**Fan5**		

1	0.00	0.97	0.87	0.87	0.00	X	P
2	0.96	0.98	0.98	0.12	0.00	X	O
3	0.99	0.99	0.98	0.51	0.98	O	O
4	0.00	0.98	0.99	0.98	0.99	X	X

(**B**) Results of a correlation-based network with a threshold of 0.40.							

**Test case**	**Credibility**					**Decision**	**Accuracy**
	**CPU1**	**Core2**	**VcoreA**	**Vbat**	**Fan5**		

1	0.00	0.97	0.87	0.87	0.00	X	P
2	0.00	0.98	0.98	0.12	0.00	X	P
3	0.99	0.99	0.98	0.73	0.73	O	O
4	0.00	0.99	0.88	0.98	0.98	X	X

(**C**) Results of a correlation-based network with a threshold of 0.52.							

**Test case**	**Credibility**					**Decision**	**Accuracy**
	**CPU1**	**Core2**	**VcoreA**	**Vbat**	**Fan5**		

1	0.34	0.67	0.50	0.34	0.01	X	O
2	0.81	0.61	0.50	0.81	0.00	X	O
3	0.99	0.99	0.50	0.73	0.73	O	O
4	0.00	0.98	0.50	0.98	0.98	X	X

(**D**) Results of a correlation-based network with a threshold of 0.55.							

**Test case**	**Credibility**					**Decision**	**Accuracy**
	**CPU1**	**Core2**	**VcoreA**	**Vbat**	**Fan5**		

1	0.87	0.97	0.50	0.87	0.00	X	O
2	0.87	0.97	0.50	0.87	0.00	X	O
3	0.98	0.99	0.50	0.88	0.98	O	O
4	0.67	0.95	0.50	0.87	0.67	O	O

(**E**) Results of a correlation-based network with a threshold of 0.62.							

**Test case**	**Credibility**					**Decision**	**Accuracy**
	**CPU1**	**Core2**	**VcoreA**	**Vbat**	**Fan5**		

1	0.84	0.84	0.50	0.50	0.00	X	O
2	0.84	0.84	0.50	0.50	0.00	X	O
3	0.61	0.81	0.50	0.50	0.81	O	O
4	0.81	0.61	0.50	0.50	0.81	O	O

(**F**) Results of a correlation-based network with a threshold of 0.90.							

**Test case**	**Credibility**					**Decision**	**Accuracy**
	**CPU1**	**Core2**	**VcoreA**	**Vbat**	**Fan5**		

1	0.84	0.84	0.50	0.50	0.50	O	X
2	0.84	0.84	0.50	0.50	0.50	O	X
3	0.84	0.84	0.50	0.50	0.50	O	O
4	0.84	0.84	0.50	0.50	0.50	O	O

**Table 9.** Diagnostic accuracy of multiple networks.

**Test case**	**(A)**	**(B)**	**(C)**	**(D)**	**(E)**	**(F)**	**(G)**	**(H)**	**(I)**	**(J)**

1	O	X	O	X	X	O	O	X	P	X
2	O	X	O	X	X	O	O	O	X	X
3	O	O	O	O	O	O	O	X	O	O
4	O	X	O	O	O	O	O	O	X	O

**Table 10.** Results of diagnostic model (C).

**Test case**	**Credibility**					**Decision**	**Accuracy**
	**CPU1**	**Core2**	**VcoreA**	**Vbat**	**Fan5**		

1	0.640	0.640	0.659	0.659	0.021	X	O
2	0.640	0.640	0.659	0.659	0.021	X	O
3	0.844	0.844	0.659	0.659	0.844	O	O
4	0.385	0.683	0.659	0.659	0.385	O	O

**Table 11.** Results of diagnostic model (I).

**Test case**	**Credibility**					**Decision**	**Accuracy**
	**CPU1**	**Core2**	**VcoreA**	**Vbat**	**Fan5**		

1	0.021	0.293	0.640	0.640	0.293	X	P
2	0.385	0.293	0.683	0.385	0.293	O	X
3	0.844	0.659	0.844	0.844	0.659	O	O
4	0.021	0.659	0.640	0.640	0.659	X	X
